# Association of toll-like receptor 2 gene polymorphisms with normal tension glaucoma

**Published:** 2009-12-26

**Authors:** Jutaro Nakamura, Akira Meguro, Masao Ota, Eiichi Nomura, Tadayuki Nishide, Kenji Kashiwagi, Fumihiko Mabuchi, Hiroyuki Iijima, Kazuhide Kawase, Tetsuya Yamamoto, Makoto Nakamura, Akira Negi, Takeshi Sagara, Teruo Nishida, Masaru Inatani, Hidenobu Tanihara, Makoto Aihara, Makoto Araie, Takeo Fukuchi, Haruki Abe, Tomomi Higashide, Kazuhisa Sugiyama, Takashi Kanamoto, Yoshiaki Kiuchi, Aiko Iwase, Shigeaki Ohno, Hidetoshi Inoko, Nobuhisa Mizuki

**Affiliations:** 1 Department of Ophthalmology, Yokohama City University School of Medicine, Yokohama, Kanagawa, Japan; 2 Department of Legal Medicine, Shinshu University School of Medicine, Matsumoto, Nagano, Japan; 3 Department of Ophthalmology, University of Yamanashi, Faculty of Medicine, Yamanashi, Japan; 4 Department of Ophthalmology, Gifu University Graduate School of Medicine, Gifu, Japan; 5 Department of Surgery, Division of Ophthalmology, Kobe University Graduate School of Medicine, Kobe, Hyogo, Japan; 6 Department of Biomolecular Recognition and Ophthalmology, Yamaguchi University School of Medicine, Ube, Yamaguchi, Japan; 7 Department of Ophthalmology and Visual Science, Graduate School of Medical Sciences, Kumamoto University, Kumamoto, Japan; 8 Department of Ophthalmology, University of Tokyo School of Medicine, Tokyo, Japan; 9 Division of Ophthalmology and Visual Science, Graduated School of Medical and Dental Sciences, Niigata University, Niigata, Japan; 10 Department of Ophthalmology and Visual Science, Kanazawa University Graduate School of Medical Science, Kanazawa, Ishikawa, Japan; 11 Department of Ophthalmology and Visual Science, Graduate School of Biomedical Sciences, Hiroshima University, Hiroshima, Japan; 12 Department of Ophthalmology, Tajimi Municipal Hospital, Tajimi, Gifu, Japan; 13 Department of Ocular Inflammation and Immunology, Hokkaido University Graduate School of Medicine, Sapporo, Hokkaido, Japan; 14 Department of Genetic Information, Division of Molecular Life Science, Tokai University School of Medicine, Isehara, Kanagawa, Japan

## Abstract

**Purpose:**

Toll-like receptor 2 (TLR2) is a transmembrane receptor that mediates immune responses to exogenous and endogenous ligands, and interacts with heat-shock proteins, which are reportedly involved in normal tension glaucoma (NTG). We investigated whether *TLR2* polymorphisms are associated with NTG.

**Methods:**

200 Japanese patients with NTG and 128 healthy Japanese controls were recruited. We genotyped five single-nucleotide polymorphisms (SNPs) in the *TLR2* gene and assessed the allele and haplotype diversities between cases and controls for all SNPs.

**Results:**

No significant differences in the frequency of *TLR2* alleles and haplotypes in the NTG cases were detected, compared with the controls.

**Conclusions:**

Our study showed no evidence for an association between *TLR2* polymorphisms and NTG. *TLR2* polymorphisms may not play an important role in NTG pathogenesis in the Japanese population.

## Introduction

Normal tension glaucoma (NTG) is a type of progressive optic neuropathy. This neuropathy, combined with normal intraocular pressure, open iridocorneal angles, and no other evidence of disease, makes NTG an insidious disease. People who suffer from NTG often show no symptoms until the disease has progressed. If the disease is diagnosed before its advance in development, it can be successfully treated via medication, surgery, or laser treatment. Yet, because of this difficulty in diagnosis, it continues to be the greater cause of total blindness in people [1-3].

The difficulty in diagnosis can be partially attributed to NTG patients exhibiting intraocular pressure levels that are classified as normal when compared with the rest of the population. Even though it is thought that intra-ocular pressure (IOP) is attributable to NTG, in reality, it does not have as much involvement in NTG as does high-tension glaucoma [4]. Aside from IOP, the development and progression of NTG have carried several other risk factors, which include ischemia, genetic predisposition, refraction, and systemic illness [5-8].

In addition, despite NTG showing many signs of having a heritable nature, identifying the genes that cause NTG remains difficult. For example, 20 different loci have been linked to primary open-angle glaucoma, which is the most common type of glaucoma [9]. Yet, only two of them have been identified as disease-causing until recently. Because the hereditary forms of glaucoma are genetically heterogeneous, detecting the genes that are susceptible to glaucoma could aid in early diagnosis and subsequent treatment.

Toll-like receptor (TLR) proteins are a family of phylogenetically conserved receptors that recognize both endogenous and exogenous. They help with innate and adaptive immunity. TLRs have emerged as a major component of the immune system. Recognition of pathogen-associated molecular patterns by TLRs activates signaling events that induce the expression of effector molecules, such as cytokines and chemokines, which control the adaptive immune responses [10,11].

In addition, studies have found that TLR polymorphisms are associated with a risk of bacterial infections and/or various diseases [12-17]. Among the TLR family members, *TLR2* and *TLR4* are the most-characterized members, and these proteins recognize heat shock protein (HSP) and lipopolysaccharide, which were previously noted as potential candidates for NTG antigens [18-21]. Yet, although TLR4 recognizes both endogenous and exogenous, TLR2 only recognizes endogenous HSPs.

Other studies show a connection between abnormal immunity and NTG, which might show that NTG is a glaucomatous condition accelerated by deviant antibodies attacking retinal tissue and causing apoptosis, or the natural death of a cell. Increased immunoactivity to bacterial hsp60, a pathogen with a similar makeup to retinal tissue, was significantly elevated in the sera of patients with NTG [19]. In addition, in groups of American patients having glaucoma, monoclonal gammopathy [20], retinal immunoglobulin deposition [21], and elevated serum antibodies titering to retinal antigens [22] (bacterial and human HSP60, HSP27, and αB-crystallin [23]) have been prevalent.

Our previous analysis indicates that *TLR4* polymorphisms have a relationship with abnormal immunity, and potentially with NTG [24]. Because TLR2 recognizes the HSPs, which are suggested to be the candidate antigen of NTG, we hypothesized that *TLR2* polymorphisms may be associated with the risk of NTG. To test this hypothesis, we performed a single-nucleotide polymorphism (SNP) analysis of *TLR2* in patients with NTG and healthy controls.

## Methods

### Subjects

The study population was comprised of a cohort of 200 unrelated Japanese patients with NTG, which included 106 women and 94 men. Their age range was 20–60 years, with a mean age of 47.3±13.9. All study patients were part of a group of 200 patients that had been previously clinically investigated at Yokohama City University, Yamanashi University, Gifu University, Kobe University, Yamaguchi University, Kumamoto University, Hokkaido University, Tokyo University, Niigata University, Kanazawa University, Hiroshima University, and Tajimi Municipal Hospital in Japan. Selected from this group for molecular genetic analysis were 200 patients who had been followed long-term. This was to ensure diagnosis of NTG, with a maximum of certainty. The criteria applied for the diagnosis of NTG were those proposed in our previous study [24]. The mean refraction value was −3.89±3.01 diopters (D), and the mean deviation observed in the Humphrey^®^ static visual field analyzer (HFA) C-30–2 program (Carl Zeiss Meditec, Oberkochen, Germany) was −9.82±7.96 dB.

Control DNA samples were obtained from 128 unrelated subjects of Japanese descent who did not have a family history of glaucoma. Control individuals were of Japanese ethnicity, age-matched (mean age 60.5±7.3), and were collected from the same geographic region as the probands. A diagnosis of glaucoma was ruled out based on IOP measurements and ophthalmoscopy of the optic disc. Written informed consent was obtained from all participants. This study was approved by the ethics committee of the Yokohama City University School of Medicine and complied with the guidelines of the Declaration of Helsinki.

### DNA preparation and *TLR2* genotype identification

Peripheral blood lymphocytes were collected, and genomic DNA was extracted from peripheral blood cells using the QIAamp DNA Blood Maxi Kit (Qiagen, Valencia, CA). *TLR2* comprises two exons and has two transcript isoforms (A and B). We evaluated five SNPs: rs1898830, rs11938228, rs3804099, rs3804100, and rs7656411. These SNPs are located within *TLR2*, including 6 kb of the predicted 3′ UTR, with minor allele frequencies >5%, according to the National Center for Biotechnology Information dbSNP and HapMap databases (Table 1). Genotyping of all SNPs was performed by TaqMan 5′ exonuclease assay using primers supplied by ABI (Applied Biosystems, Foster City, CA). The probe fluorescence signal was detected using the TaqMan Assay for Real-Time PCR (7500 Real Time PCR System; Applied Biosystems), following the manufacturer’s instructions.

**Table 1:** Allele frequencies of SNPs of *TLR2* among NTG patients and controls.

**dbSNP**	**Alleles (1/2)**	**Position (bp)**	**Gene location**	**Minor allele frequency, n (%)**		**p**
				**Cases (n=200)**	**Controls (n=128)**	
rs1898830	A/G	154,827,903	Intron	193 (48.3)	122 (47.7)	0.881
rs11938228	C/A	154,841,396	Intron	187 (46.8)	119 (46.5)	0.947
rs3804099	T/C	154,844,106	Exon	124 (31.0)	71 (27.7)	0.372
rs3804100	T/C,	154,844,859	Exon	115 (28.8)	63 (24.6)	0.245
rs7656411	G/T	154,847,105	3’UTR	180 (45.0)	108 (42.2)	0.479

In the "Alleles" column, 1 indicates the major allele and 2 indicates the minor allele. Position is distance from short arm telomere. bp, base pairs. p-values were caluculated by χ^2^ test 2×2 contingency table.

### Statistical analysis

The Hardy-Weinberg equilibrium was tested for each SNP among controls. Differences in allele frequency between case and control were assessed by χ2 test and Fisher’s exact test. The program Haploview 3.32 was used to compute pairwise linkage disequilibrium (LD) statistics [25]. Standardized disequilibrium (D’) was plotted. LD blocks were defined according to the criteria of Gabriel et al. [26]. Haplotype frequencies were estimated using an accelerated expectation-maximization algorithm similar to the partition-ligation-expectation-maximization method [27]. All p-values were derived from a 2-sided test, and p-values < 0.05 were considered statistically significant.

## Results

**Figure 1:**
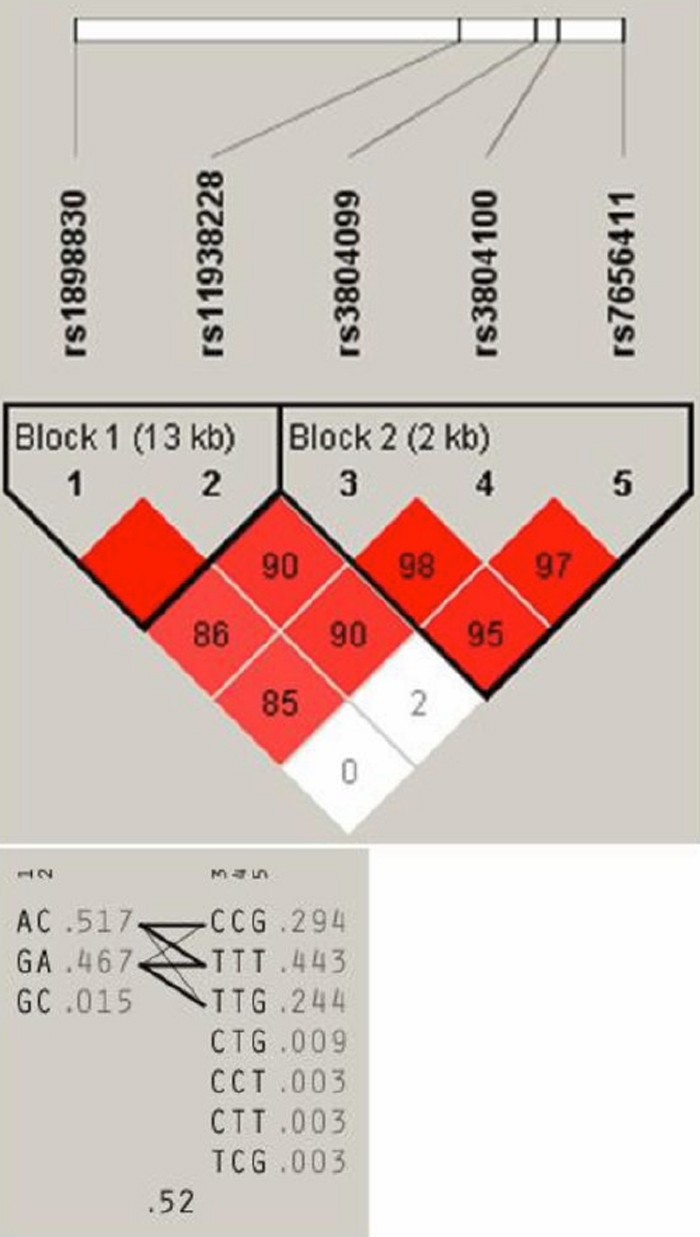
**A**: Linkage disequilibrium plot of five SNPs of the *TLR2* gene in NTG patients and controls. A schematic of *TLR2* is shown as a black line, with boxes representing its three exons. The locations of the selected SNPs are indicated by the dotted lines. The D’ value corresponding to each SNP pair is expressed as a percentage and shown within the respective square. Higher D’ values are indicated in brighter red. **B**: Haplotype structure and diversity of *TLR2*. Common haplotypes of the two blocks are listed. Haplotype frequencies observed in NTG patients and controls are given in parentheses. The line thickness reflects the frequency of adjacent block haplotype distribution (thick lines, >10%; thin lines, 1%–10%).

Five SNPs in *TLR2* were genotyped. Genotype distributions of all SNPs in controls exhibited Hardy-Weinberg equilibrium (data not shown), and the minor allele frequencies of all SNPs were over 5% in controls (Table 1). We determined that the gene region could be divided into two haplotype blocks, with substantial LD among the SNPs of both blocks (block 1: D’ ≥0.96; block 2: D’ ≥0.98; Figure 1A).

The allele frequencies of the five SNPs in cases and controls are listed in Table 1. No statistically significant association was observed for any of the SNPs between cases and controls (p>0.05). Haplotypes for each block and their frequencies are listed in Table 2. Two common haplotypes were observed within block 1, and three in block 2 (frequency >10%). We observed four common haplotypes of the five SNPs that extended across the two blocks (Figure 1B). As we observed, the frequencies of minor haplotypes in block 2 and two blocks, CTG and ACCTG, were decreased in cases, compared with the controls. However, this decrease did not reach statistical significance when evaluated using Bonferoni’s correction. No other significant differences in common haplotype frequencies between cases and controls were detected.

**Table 2:** Haplotype frequencies of tag SNPs of *TLR2* among NTG patients and controls.

**Haplotype**	**Frequency, %**		**p**	**Pc**
	**Cases (n=200)**	**Controls (n=128)**		
Block 1 (tag SNPs rs1898830, rs11938228)				
AC	51.8	52.3	0.88	
GA	46.8	46.5	0.95	
GC	1.5	1.2	0.72	
Block 2 (tag SNPs rs3804099, rs3804100, and rs7656411)				
TTT	44.3	41.7	0.50	
CCG	29.4	24.1	0.14	
TTG	24.4	30.6	0.081	
CTG	1.0	3.1	0.047	0.19
Block 1+2 (tag SNPs rs1898830, rs11938228, rs3804099, rs3804100, and rs7656411)				
GATTG	22.9	29.0	0.081	
ACCCG	26.6	21.2	0.12	
ACTTT	22.2	26.0	0.25	
GATTT	22.1	15.7	0.043	0.34
ACCTG	1.0	3.1	0.044	0.35
GACCG	1.7	1.8	0.93	
ACTTG	1.0	1.4		
GCCCG	1.0	1.2	0.83	

Haplotypes with frequency less than 1% are not listed. p values were caluculated by χ^2^ test 2×2 contingency table. We corrected these p values (*P*c) for multiple testing by Bonferoni's correction.

## Discussion

The purpose of this study was to determine whether or not *TLR2* polymorphisms are associated with the risk of NTG, based on recent findings of increased immunoactivity in TLR2, after learning that TLR4 has a relationship with NTG. Previously, we analyzed the relationship between *TLR4* polymorphisms and the development of NTG [24]. This analysis infers that the polymorphisms of rs7037117, located in the 3′-untranslated lesion, have a strong association with the clinical characteristics of NTG. To compare these results with our previous results, we genotyped five single-nucleotide polymorphisms (SNPs) in *TLR2*, and assessed the allele and haplotype diversities between cases and controls for all SNPs. Here, we report a lack of association between *TLR2* polymorphisms and NTG in Japanese patients, suggesting that the abnormal function found and *TLR2* polymorphisms do not contribute to NTG, due to the results of the test comparing subjects and controls.

Recently, it has been suggested that the immune system and heat shock proteins (HSPs) play important roles in glaucoma [18]. HSPs are highly immunogenic molecules that are widely distributed in nature; they perform important functions relating to the folding and assembly of protein complexes. Human HSPs are expressed on cell membranes in response to stress, such as physiologic shock and microbial challenge. Wax and Tezel et al. [19] observed that NTG patients have increased serum immunoreactivity to bacterial and human HSP60. They also showed that direct application of antibodies to HSPs resulted in neuronal apoptosis, and NTG patients had higher titers of antibodies to HSPs, including HSP27 and HSP60, compared both to patients with high IOP and to healthy controls [23]. Furthermore, they observed increased expression of HSP27 and HSP60 in the glaucomatous retina and/or optic nerve head, and proposed that immune regulation of these HSPs is an important component of glaucomatous optic neuropathy [18].

In retrospect, TLR4 has the ability to distinguish between exogenous ligands and endogenous ligands. Many different exogenous and endogenous ligands bind to TLR4, thereby activating an innate immune response. For example, TLR4 can distinguish between the two different types of HSP60 (self and *Chlamydia pneumoniae*). Yet TLR2 cannot process the bacterial (foreign) form of HSP60. Bacterial HSP60 was shown to have sparked serum immunoreactivity in previous studies [19], and thus bears significance when introduced to TLR2 and TLR4. Yet, as was deduced from our study, there was not a significant relationship between TLR2 and NTG. Therefore, because TLR2 cannot recognize bacterial HSP60, perhaps the abnormal recognition of HSP60 by TLR4 is the beginning of normal autoimmunity in NTG. Studies to confirm this may prove essential to determining autoimmune effects in glaucoma optic neuropathy.

In NTG patients, due to the delayed ability to diagnose the progression of the disease, it was thought that identifying a susceptibility gene and elucidating pathogenic mechanisms was the best course of action to take. Through other studies, it has been concluded that polymorphisms in TLRs are associated with blunted immune responses to microbial pathogens. Our advanced analysis indicates that the common polymorphisms in *TLR2* are not associated with NTG in Japanese patients. Though the possibility of *TLR2* polymorphisms being primarily associated with the pathogenesis of NTG is deemed low, the TLR2 ligands, HSP, and lipopolysaccharide, may act as risk factors for NTG, and changes in cytokine secretion induced by the ligands may contribute to the induction of pathological immune reaction in NTG [28-33]. In conclusion, further studies are essential for a more detailed investigation of TLR signaling pathways related to the ligands in NTG patients.
